# Investigating HER2‐Low in Early Breast Cancer: Prognostic Implications and Age‐Related Prognostic Stratification

**DOI:** 10.1002/cam4.70637

**Published:** 2025-02-13

**Authors:** Endong Chen, Chen Chen, Yingying Chen, Jie You, Nan Chen, Shenlin Xu, Qingxuan Wang, Yefeng Cai, Xiaoqu Hu, Quan Li

**Affiliations:** ^1^ Department of Breast Surgery The First Affiliated Hospital of Wenzhou Medical University Zhejiang China; ^2^ The First School of Medicine, School of Information and Engineering Wenzhou Medical University Zhejiang China; ^3^ Department of Colorectal and Anal Surgery The First Affiliated Hospital of Wenzhou Medical University Zhejiang China; ^4^ Department of Thyroid Surgery The First Affiliated Hospital of Wenzhou Medical University Zhejiang China

**Keywords:** age, breast cancer, Chinese population, HER2‐low, prognostic stratification

## Abstract

**Background:**

Recent studies about human epidermal growth factor receptor 2 (HER2)‐low values have garnered great interest among oncologists. We aimed to investigate whether HER2‐low impacts the prognosis of early‐stage breast cancer overall and in specific subgroups, explore differences in clinicopathologic markers, and examine the role of age in HER2‐low prognostic stratification.

**Materials & Methods:**

We conducted a retrospective analysis of 6920 HER2‐negative breast cancer patients from the First Affiliated Hospital of Wenzhou Medical University (2010–2022). The study focused on the impact of HER2‐low status (immunohistochemistry +1 or +2, in situ hybridization not amplified) on overall survival (OS), considering the age at diagnosis.

**Results:**

Generally, HER2‐low status correlated with less aggressive cancer indicators. No significant prognostic differences were observed between HER2‐low and HER2‐0 in the entire cohort, HR‐positive, and HR‐negative groups. However, in TNBC patients aged ≥ 65, HER2‐low correlated with significantly better OS (HR = 0.45, 95% CI 0.24–0.83, *p* = 0.011), a finding consistent after multivariable adjustment (HR = 0.34, 95% CI 0.14–0.80, *p* = 0.014). In other subgroups, prognosis did not significantly correlate with HER2 status. The combination of HER2‐low status and age plays a key role in prognostic stratification in TNBC. Patients aged ≥ 65 with HER2‐0 had considerably poorer prognoses compared to other subgroups.

**Conclusion:**

This extensive retrospective study demonstrates that HER2‐low status cannot serve as an independent prognostic factor in the entire cohort, nor in the HR‐positive and HR‐negative groups individually. However, the combined factors of HER2‐low status and age may indicate a potential contribution to the prognostic stratification of TNBC.

## Graphical abstract

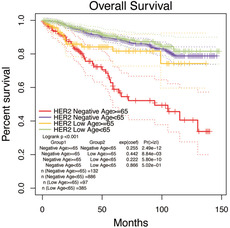

**Abbreviations**
ER: estrogenoestrogen receptor
HER2: human epidermal growth factor receptor 2
HR: hormone receptor
IQR: interquartile range
OS: overall survival
PR: progesterone receptor

## 1 Introduction

In 2022, breast cancer led to 670,000 deaths globally, becoming the most common cancer among women in 157 of 185 countries [1]. According to 2024 data, breast cancer is the second leading cause of cancer death among women in the United States, following lung cancer, with 42,250 expected deaths [2]. Breast cancer is classified into four subtypes based on pathologic receptor types: Luminal A, Luminal B, HER2‐positive (human epidermal growth factor receptor 2 positive), and TNBC (triple‐negative breast cancer). This classification guides different treatment modalities for each subtype [3, 4]. The discovery of the HER2 receptor and subsequent drug developments have significantly advanced breast cancer research and treatment [5, 6]. About 15%–20% of breast cancer patients are HER2 positive, characterized by HER2 protein overexpression on an immunohistochemistry assay (IHC 3+ score) or ERBB2 gene amplification on in situ hybridization (ISH) [7], while the remaining are HER2 negative, categorized into Luminal and TNBC subclasses based on hormone receptor (HR) status.

Recent discoveries have shown the efficacy of antibody‐drug conjugates (ADCs) [8] such as trastuzumab deruxtecan (T‐DXd), trastuzumab duocarmazine, and disitamab vedotin in treating patients with low HER2 expression (IHC score of 1+ or 2+ without HER2 gene amplification) [9, 10, 11, 12], although anti‐HER2 monoclonal antibodies targeting HER2 receptors did not bring meaningful clinical benefits [13, 14]. Novel ADCs offer a promising therapeutic option for advanced breast cancer with HER2‐low, garnering significant interest among oncologists. About 65% of Luminal‐type and 37% of TNBC patients can be classified as HER2‐low [15]. This substantial proportion underscores the clinical importance of studying HER2‐low breast cancer.

Current research on HER2‐low breast cancer encompasses advanced breast cancer treatment, prognostic value, biological differences, clinical trial design, and pathologic diagnosis [16, 17]. Our study focuses on the prognostic value of HER2‐low. Conclusions about whether HER2‐low can serve as an independent prognostic factor in breast cancer are inconsistent. Some studies support its value in prognostic stratification [18, 19, 20, 21], whereas the majority of studies have concluded that there is no significant prognostic difference between HER2‐low and HER2‐0 [15, 22, 23, 24, 25, 26, 27]. The latest ESMO expert consensus reveals that a large majority (94%, 30/32) of experts see no significant prognostic difference between HER2‐low and HER2‐0 breast cancer [16]. However, whether there is a prognostic difference between HER2‐low and HER2‐0 in a more specific subgroup remains underexplored, with few studies addressing this [27, 28]. This study was conducted to examine clinicopathological differences between HER2‐low and HER2‐0, analyze HER2‐low's prognostic difference in our dataset, and explore its prognostic role across different age groups.

## 2 Methods

### 2.1 Study Design and Participants

This is a retrospective cohort that enrolled 9593 HER2‐negative patients diagnosed with primary invasive breast cancer between 2010 and 2022 at the First Affiliated Hospital of Wenzhou Medical University (WMU), China. Patients had to meet the following inclusion criteria: underwent breast surgery, histologically proven invasive breast cancer, and clinical stage I–III. Patients with HER2‐positive BC (IHC + 3 or IHC + 2 FISH/ISH amplified, *n* = 2543) or unknown HER2 status were excluded. Other exclusion criteria included insufficient data about tumor pathological characteristics or treatment received. This resulted in a final study cohort of 6920 patients, representing roughly 72.1% of the initially identified group. Most patients presented with T1 (62%), N0 stage (63%), grade I (45%), ER‐positive (77%) and HER2‐0 disease (52%). The median age was 51 years [interquartile range (IQR) 44–61]. Median follow‐up of this population was 41 (IQR 18–73) months.

Information from the First Affiliated Hospital of WMU cohort contained age at diagnosis, menopausal status, estrogen receptor (ER), progesterone receptor (PR), and HR (HR was deemed positive if at least 1% of invasive tumor cells exhibited immunostaining for ER or PR).

For the HER2 status assessment, HER2 IHC slides were rescored according to the latest 2018 ASCO/CAP guideline [7]. HER2‐positive is defined as HER2 IHC 3+ or IHC 2+ and ISH+; HER2‐low is defined as IHC 1+ or IHC 2+ and ISH−; HER2‐0 is defined as IHC 0.

The dataset also included histological grade, tumor size (cm), lymph metastasis, AJCC stage (adhered to the 8th edition of the tumor, node, and metastasis classification system), metastasis (de novo metastatic disease after diagnosis, metastasis at first diagnosis was excluded), type of adjuvant therapy (hormone therapy, chemotherapy, and radiation therapy). Through the hospital's records database and the Wenzhou Health Service Platform, we obtained the death registration information for patients. Analyses were censored on 31 December 2022 to allow for delay in reporting of vital status. When assessing 5‐year overall survival, follow‐up time was defined as the time from breast cancer diagnosis to last follow‐up, death or 5 years after diagnosis, whichever came first.

### 2.2 Statistical Analysis

Categorical variables were compared using the Pearson chi‐square test and Fisher's exact test. Comparison of median and IQR of continuous variables in nonparametric independent samples was performed using the Wilcoxon–Mann–Whitney test. Kaplan–Meier (K–M) analysis was used to estimate the 5‐year survival probability, and the log‐rank test was used to compare the differences between K‐M curves of patients in various groups. Multivariate analyses utilized Cox proportional hazards regression models to calculate adjusted hazard ratios (HRs) with 95% confidence intervals (CI). All analyses were performed in R version 4.2.2 (The R Foundation, Vienna, Austria). A *p*‐value threshold of 0.05 was defined as statistically significant.

## 3 Results

### 3.1 Clinicopathological Characteristics of the Patients According to HER2 Status

Clinicopathological characteristics for the entire cohort, stratified by HR status, comparing differences between patients with HER2‐low and HER2‐0 breast cancer were summarized in Table 1. Among the entire cohort, 5404 (78.3%) presented with HR‐positive breast cancer, while 1500 (21.7%) had triple‐negative breast cancer (TNBC). HER2‐low breast cancer had a higher proportion of HR‐positive patients (85.5%) compared to HER2‐0 (71.6%, *p* < 0.001), with similar results for ER‐positive and PR‐positive proportions (84.5% vs. 69.9%, *p* < 0.001; 74.8% vs. 63.0%, *p* < 0.001). A greater percentage of patients with HER2‐low breast cancer underwent hormone therapy (72.2% vs. 61.2%, *p* < 0.001). Furthermore, HER2‐low breast cancer was associated with a lower percentage of grade 3 tumors than the HER2‐0 group (25.4% vs. 33.6%, *p* < 0.001), similar to HR‐negative patients (59.5% vs. 68.7%, *p* = 0.001). HER2‐low patients exhibited lower instances of bone metastasis (8.1% vs. 9.8%, *p* = 0.013) for the entire cohort.

**TABLE 1:** Demographics and relationship between HER2 and clinicopathological characteristics of Breast cancer.

	Total	HER2‐negative	HER2‐low	
	*n* (%)	*n* (%)	*n* (%)	*p*
Entire cohort				
*n* (%)	6920			
Sex (male)	49/6920 (0.7)	22/3593 (0.6)	27/3327 (0.8)	0.323
Age at diagnosis, *n*	6920/6920 (100.0)	3593/3593 (100.0)	3327/3327 (100.0)	
Median (IQR), years	51 (44, 61)	51 (44, 61)	52 (44, 61)	0.123
Age ≥ 65 years	1173/6920 (17.0)	610/3593 (17.0)	563/3327 (16.9)	0.951
Post‐menopausal	2895/4397 (65.8)	1489/2278 (65.4)	1406/2119 (66.4)	0.490
Tumor size, *n*	6322/6920 (91.4)	3261/3593 (90.8)	3061/3327 (92.0)	0.066
Median (IQR), cm	2.00 (1.30, 2.80)	2.00 (1.30, 3.00)	2.00 (1.30, 2.50)	0.376
Tumor size ≥ 2.0 cm	3480/6322 (55.0)	1809/3261 (55.5)	1671/3061 (54.6)	0.480
Tumor stage III	1134/6432 (17.6)	564/3322 (17.0)	570/3110 (18.3)	0.156
HR Positive	5404/6904 (78.3)	2570/3588 (71.6)	2834/3316 (85.5)	< 0.001
ER Positive	5304/6897 (76.9)	2507/3585 (69.9)	2797/3312 (84.5)	< 0.001
PR Positive	4728/6882 (68.7)	2251/3572 (63.0)	2477/3310 (74.8)	< 0.001
KI67 ≥ 20	4282/6790 (63.1)	2252/3514 (64.1)	2030/3276 (62.0)	0.070
Grade 3	1676/5694 (29.4)	945/2813 (33.6)	731/2881 (25.4)	< 0.001
Lymph metastasis	2535/6920 (36.6)	1263/3593 (35.2)	1272/3327 (38.2)	0.008
Distant metastasis	1197/6920 (17.3)	645/3593 (18.0)	552/3327 (16.6)	0.135
Liver metastasis	229/6920 (3.3)	118/3593 (3.3)	111/3327 (3.3)	0.904
Bone metastasis	621/6920 (9.0)	352/3593 (9.8)	269/3327 (8.1)	0.013
Brain metastasis	233/6920 (3.4)	127/3593 (3.5)	106/3327 (3.2)	0.422
Lung metastasis	251/6920 (3.6)	133/3593 (3.7)	118/3327 (3.5)	0.731
Bilateral	146/6919 (2.1)	83/3592 (2.3)	63/3327 (1.9)	0.228
Chemotherapy	4638/6920 (67.0)	2444/3593 (68.0)	2194/3327 (65.9)	0.066
Hormone Therapy	4600/6920 (66.5)	2199/3593 (61.2)	2401/3327 (72.2)	< 0.001
Radiation therapy	2183/6920 (31.5)	1133/3593 (31.5)	1050/3327 (31.6)	0.981
Follow‐up time	6920/6920 (100.0)	3593/3593 (100.0)	3327/3327 (100.0)	
Median (IQR), months	41 (18, 73)	42 (18, 80)	41 (17, 69)	< 0.001
Five‐year OS	91.4 (90.5–92.3)	91.0 (89.8–92.2)	91.8 (90.6–93.1)	0.091
HR positive				
*n* (%)	5404			
Sex (male)	49/5404 (0.9)	22/2570 (0.9)	27/2834 (1.0)	0.708
Age at diagnosis, *n*	5404/5404 (100.0)	2570/2570 (100.0)	2834/2834 (100.0)	
Median (IQR), years	51 (44, 61)	51 (44, 62)	52 (44, 61)	0.367
Age ≥ 65 years	942/5404 (17.4)	478/2570 (18.6)	464/2834 (16.4)	0.031
Post‐menopausal	2255/3460 (65.2)	1081/1645 (65.7)	1174/1815 (64.7)	0.525
Tumor size, *n*	4938/5404 (91.4)	2320/2570 (90.3)	2618/2834 (92.4)	0.006
Median (IQR), cm	2.00 (1.20, 2.50)	2.00 (1.20, 2.50)	2.00 (1.30, 2.50)	0.230
Tumor size ≥ 2.0 cm	2550/4938 (51.6)	1173/2320 (50.6)	1377/2618 (52.6)	0.153
Tumor stage III	884/5030 (17.6)	401/2367 (16.9)	483/2663 (18.1)	0.266
KI67 ≥ 20	2942/5312 (55.4)	1313/2509 (52.3)	1629/2803 (58.1)	< 0.001
Grade 3	876/4468 (19.6)	387/1998 (19.4)	489/2470 (19.8)	0.720
Lymph metastasis	2012/5404 (37.2)	914/2570 (35.6)	1098/2834 (38.7)	0.016
Distant metastasis	870/5404 (16.1)	422/2570 (16.4)	448/2834 (15.8)	0.541
Liver metastasis	174/5404 (3.2)	85/2570 (3.3)	89/2834 (3.1)	0.728
Bone metastasis	463/5404 (8.6)	235/2570 (9.1)	228/2834 (8.0)	0.150
Brain metastasis	147/5404 (2.7)	70/2570 (2.7)	77/2834 (2.7)	0.988
Lung metastasis	150/5404 (2.8)	63/2570 (2.5)	87/2834 (3.1)	0.167
Bilateral	101/5403 (1.9)	50/2569 (1.9)	51/2834 (1.8)	0.691
Chemotherapy	3347/5404 (61.9)	1556/2570 (60.5)	1791/2834 (63.2)	0.045
Hormone Therapy	4508/5404 (83.4)	2143/2570 (83.4)	2365/2834 (83.5)	0.948
Radiation therapy	1672/5404 (30.9)	783/2570 (30.5)	889/2834 (31.4)	0.474
Follow‐up time	5404/5404 (100.0)	2570/2570 (100.0)	2834/2834 (100.0)	
Median (IQR), months	42 (18, 73)	44 (19, 82)	41 (18, 69)	< 0.001
Five‐year OS	92.7 (91.8–93.7)	93.1 (91.8–94.4)	92.3 (91.0–93.7)	0.843
HR negative				
*n* (%)	1500			
Sex (male)	0/1500 (0.0)	0/1018 (0.0)	0/482 (0.0)	> 0.999
Age at diagnosis, *n*	1500/1500 (100.0)	1018/1018 (100.0)	482/482 (100.0)	
Median (IQR), years	52 (45, 60)	51 (44, 59)	55 (47, 62)	< 0.001
Age ≥ 65 years	229/1500 (15.3)	132/1018 (13.0)	97/482 (20.1)	< 0.001
Post‐menopausal	635/927 (68.5)	405/629 (64.4)	230/298 (77.2)	< 0.001
Tumor size, *n*	1373/1500 (91.5)	938/1018 (92.1)	435/482 (90.2)	0.219
Median (IQR), cm	2.00 (1.50, 3.00)	2.00 (1.50, 3.00)	2.00 (1.50, 3.00)	0.379
Tumor size ≥ 2.0 cm	925/1373 (67.4)	634/938 (67.6)	291/435 (66.9)	0.799
Tumor stage III	249/1391 (17.9)	162/952 (17.0)	87/439 (19.8)	0.205
KI67 ≥ 20	1340/1477 (90.7)	939/1004 (93.5)	401/473 (84.8)	< 0.001
Grade 3	800/1219 (65.6)	558/812 (68.7)	242/407 (59.5)	0.001
Lymph metastasis	521/1500 (34.7)	347/1018 (34.1)	174/482 (36.1)	0.444
Distant metastasis	324/1500 (21.6)	222/1018 (21.8)	102/482 (21.2)	0.777
Liver metastasis	54/1500 (3.6)	32/1018 (3.1)	22/482 (4.6)	0.168
Bone metastasis	156/1500 (10.4)	116/1018 (11.4)	40/482 (8.3)	0.067
Brain metastasis	86/1500 (5.7)	57/1018 (5.6)	29/482 (6.0)	0.745
Lung metastasis	101/1500 (6.7)	70/1018 (6.9)	31/482 (6.4)	0.748
Bilateral	45/1500 (3.0)	33/1018 (3.2)	12/482 (2.5)	0.425
Chemotherapy	1285/1500 (85.7)	885/1018 (86.9)	400/482 (83.0)	0.042
Hormone Therapy	83/1500 (5.5)	53/1018 (5.2)	30/482 (6.2)	0.421
Radiation therapy	507/1500 (33.8)	348/1018 (34.2)	159/482 (33.0)	0.647
Follow‐up time	1500/1500 (100.0)	1018/1018 (100.0)	482/482 (100.0)	
Median (IQR), months	37 (16, 73)	37 (16, 75)	38 (16, 71)	0.976
Five‐year OS	86.5 (84.2–88.8)	85.3 (82.5–88.3)	88.9 (85.3–92.5)	0.166

Abbreviations: ER, estrogen receptor; HER2, human epidermal growth factor receptor 2; HR, hormone receptor; IQR, interquartile range; *n*, total number; OS, overall survival; PR, progesterone receptor.

Among patients with HR‐positive breast cancer, the median age at diagnosis did not differ significantly between HER2‐low and HER2‐0 groups (*p* = 0.367); however, fewer HER2‐low patients were over the age of 65 (16.4% vs. 18.6%, *p* = 0.031). Conversely, HER2‐low tumors within TNBC patients had a higher median diagnosis age compared to HER2‐0 tumors (55 vs. 51, *p* < 0.001), with a greater proportion of patients aged over 65 (20.1% vs. 13.0%, *p* < 0.001). The proportion of postmenopausal women was significantly higher in the HER2‐low group within the HR‐negative group (77.2% vs. 64.4%, *p* < 0.001).

For HR‐positive breast cancer, KI67 ≥ 20 was more prevalent in the HER2‐low group, whereas it was less common in TNBC (58.1% vs. 52.3%, *p* < 0.001 and 84.8% vs. 93.5%, *p* < 0.001, respectively). There was a higher proportion of lymph metastasis in the HER2‐low group among HR‐positive breast cancer patients (38.7% vs. 35.6%, *p* = 0.016).

### 3.2 The Role of HER2‐Low in the Overall Survival of Breast Cancer

**FIGURE 1:**
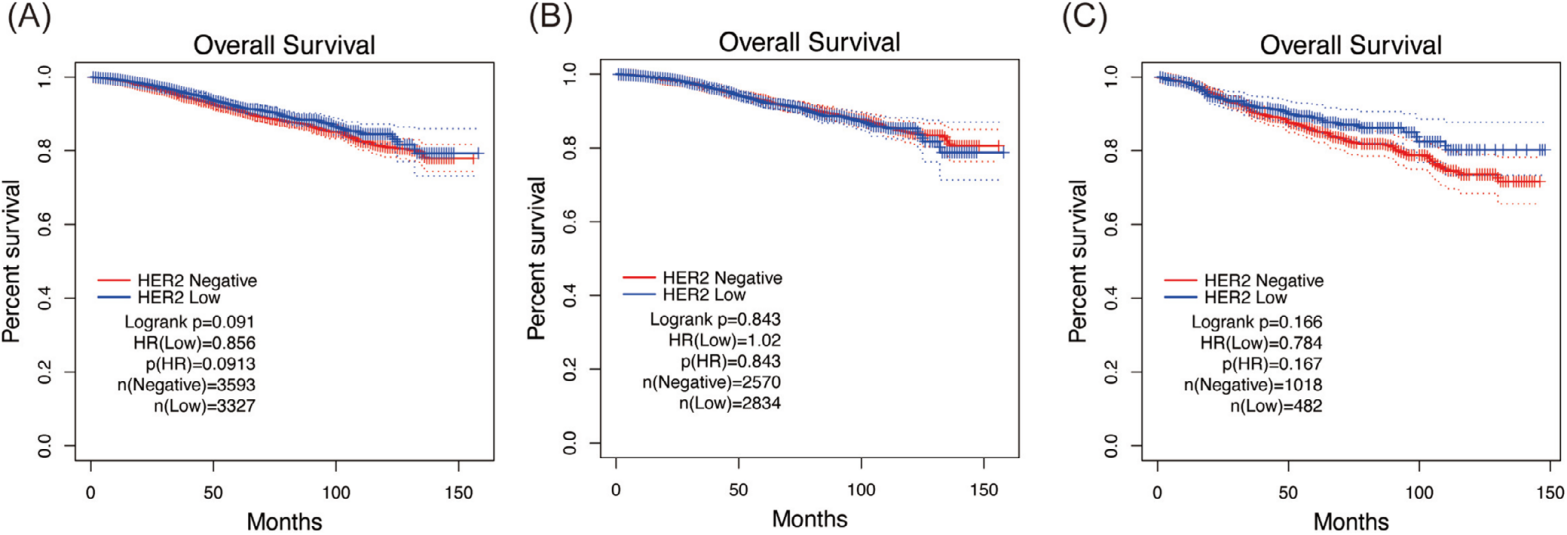
Kaplan–Meier analyses of patient survival probability associated with HER2 status in breast cancer. (A) Entire cohort. (B) HR‐positive. (C) HR‐negative.

The median follow‐up time was 41 months (IQR, 18–73 months) and during the follow‐up period, 487 cases of death from any cause in all patients were observed for the entire cohort. Kaplan–Meier survival curve analysis revealed no significant differences in overall survival (OS) between HER2‐low and HER2‐0 groups across the entire cohort (log‐rank *p* = 0.091), as well as in HR‐positive breast cancer and TNBC subgroups (*p* = 0.843 and 0.167, respectively) (Figure 1a–c). The univariate analyses of HER2 status show a similar result, and these persisted after multivariate adjustment for patient age at diagnosis, AJCC stage, KI67 status, grade, hormone therapy, and chemotherapy (Table 3). This suggests that considering HER2 status alone, there might be no prognostic difference between patients with low HER2 expression and those who are HER2‐0.

### 3.3 Differentiation of HER2‐Low Related Clinicopathological of Breast Cancer by the Age

Given the observed age differences between HER2‐low and HER2‐0 groups after HR subgroup differentiation, we aim to further investigate the correlations between HER2 status, age, and clinicopathological characteristics.

Overall, subdividing the cohort into age ≥ 65 and < 65 groups revealed a significant correlation between HER2‐low status and less aggressive clinicopathological breast cancer outcomes (Table 2). In the entire cohort, HER2‐low status correlated with a higher frequency of HR‐positive patients (*p* < 0.001 for age < 65 group), lower rates of KI67 ≥ 20 (*p* = 0.013 for age < 65 group), lower incidence of grade 3 tumors (*p* < 0.001 for age < 65 group), and reduced distant and bone metastases (*p* = 0.020 for age ≥ 65 group). In the TNBC cohort, HER2‐low status correlated with lower rates of KI67 ≥ 20 (*p* = 0.015 and *p* < 0.001 for each age group), lower incidence of grade 3 tumors (*p* = 0.020 and *p* = 0.023 for each age group, respectively) and reduced bone distant metastases (*p* = 0.006 for age ≥ 65 group).

**TABLE 2:** Relationship between HER2 and clinicopathological characteristics of Breast cancer with respect to the Age.

Characteristics	Age ≥ 65 years			Age < 65 years		
	HER2‐negative	HER2‐low		HER2‐negative	HER2‐low	
	*n* (%)	*n* (%)	*p*	*n* (%)	*n* (%)	*p*
Entire cohort						
*n* (%)						
Sex (male)	12/610 (2.0)	14/563 (2.5)	0.546	10/2983 (0.3)	13/2764 (0.5)	0.418
Age at diagnosis, *n*	610/610 (100.0)	563/563 (100.0)		2983/2983 (100.0)	2764/2764 (100.0)	
Median (IQR), years	71.0 (67.0, 76.0)	69.0 (67.0, 74.0)	0.006	49 (43, 55)	49 (43, 56)	0.019
Age ≥ 65 years	610/610 (100.0)	563/563 (100.0)	> 0.999	0/2983 (0.0)	0/2764 (0.0)	> 0.999
Post‐menopausal	597/598 (99.8)	549/549 (100.0)	> 0.999	892/1680 (53.1)	857/1570 (54.6)	0.394
Tumor size, *n*	546/610 (89.5)	516/563 (91.7)		2715/2983 (91.0)	2545/2764 (92.1)	
Median (IQR), cm	2.00 (1.50, 3.00)	2.00 (1.50, 3.00)	0.199	2.00 (1.30, 2.50)	2.00 (1.30, 2.50)	0.675
Tumor size ≥ 2.0 cm	352/546 (64.5)	303/516 (58.7)	0.054	1457/2715 (53.7)	1368/2545 (53.8)	0.949
Tumor stage III	103/554 (18.6)	100/521 (19.2)	0.801	461/2768 (16.7)	470/2589 (18.2)	0.148
HR Positive	478/610 (78.4)	464/561 (82.7)	0.061	2092/2978 (70.2)	2370/2755 (86.0)	< 0.001
ER Positive	472/609 (77.5)	458/561 (81.6)	0.080	2035/2976 (68.4)	2339/2751 (85.0)	< 0.001
PR Positive	410/607 (67.5)	384/560 (68.6)	0.707	1841/2965 (62.1)	2093/2750 (76.1)	< 0.001
KI67 ≥ 20	334/600 (55.7)	325/554 (58.7)	0.304	1918/2914 (65.8)	1705/2722 (62.6)	0.013
Grade 3	126/447 (28.2)	111/480 (23.1)	0.077	819/2366 (34.6)	620/2401 (25.8)	< 0.001
Lymph metastasis	213/610 (34.9)	204/563 (36.2)	0.638	1050/2983 (35.2)	1068/2764 (38.6)	0.007
Distant metastasis	123/610 (20.2)	86/563 (15.3)	0.029	522/2983 (17.5)	466/2764 (16.9)	0.521
Liver metastasis	22/610 (3.6)	11/563 (2.0)	0.087	96/2983 (3.2)	100/2764 (3.6)	0.404
Bone metastasis	66/610 (10.8)	39/563 (6.9)	0.020	286/2983 (9.6)	230/2764 (8.3)	0.093
Brain metastasis	18/610 (3.0)	10/563 (1.8)	0.188	109/2983 (3.7)	96/2764 (3.5)	0.712
Lung metastasis	28/610 (4.6)	17/563 (3.0)	0.162	105/2983 (3.5)	101/2764 (3.7)	0.785
Bilateral	18/610 (3.0)	8/563 (1.4)	0.075	65/2982 (2.2)	55/2764 (2.0)	0.615
Chemotherapy	240/610 (39.3)	221/563 (39.3)	0.975	2204/2983 (73.9)	1973/2764 (71.4)	0.033
Hormone therapy	413/610 (67.7)	394/563 (70.0)	0.400	1786/2983 (59.9)	2007/2764 (72.6)	< 0.001
Radiation therapy	105/610 (17.2)	86/563 (15.3)	0.369	1028/2983 (34.5)	964/2764 (34.9)	0.741
Follow‐up time	610/610 (100.0)	563/563 (100.0)		2983/2983 (100.0)	2764/2764 (100.0)	
Median (IQR), months	38 (17, 70)	31 (15, 60)	0.004	43 (18, 82)	42 (18, 71)	0.002
Five‐year OS	79.8 (75.7–84.1)	80.1 (75.5–84.9)	0.411	93.3 (92.1–94.5)	94.1 (92.8–95.3)	0.241
HR positive						
*n* (%)						
Sex (male)	12/478 (2.5)	14/464 (3.0)	0.635	10/2092 (0.5)	13/2370 (0.5)	0.743
Age at diagnosis, *n*	478/478 (100.0)	464/464 (100.0)		2092/2092 (100.0)	2370/2370 (100.0)	
Median (IQR), years	71.0 (67.0, 76.0)	69.0 (67.0, 74.0)	0.003	48 (43, 55)	49 (43, 56)	0.330
Age ≥ 65 years	478/478 (100.0)	464/464 (100.0)	> 0.999	0/2092 (0.0)	0/2370 (0.0)	> 0.999
Post‐menopausal	465/466 (99.8)	450/450 (100.0)	> 0.999	616/1179 (52.2)	724/1365 (53.0)	0.690
Tumor size, *n*	428/478 (89.5)	427/464 (92.0)		1892/2092 (90.4)	2191/2370 (92.4)	
Median (IQR), cm	2.00 (1.50, 3.00)	2.00 (1.50, 3.00)	0.467	1.80 (1.20, 2.50)	2.00 (1.20, 2.50)	0.069
Tumor size ≥ 2.0 cm	260/428 (60.7)	243/427 (56.9)	0.254	913/1892 (48.3)	1134/2191 (51.8)	0.026
Tumor stage III	79/434 (18.2)	82/433 (18.9)	0.781	322/1933 (16.7)	401/2230 (18.0)	0.261
KI67 ≥ 20	219/471 (46.5)	251/458 (54.8)	0.011	1094/2038 (53.7)	1378/2345 (58.8)	< 0.001
Grade 3	52/336 (15.5)	70/397 (17.6)	0.435	335/1662 (20.2)	419/2073 (20.2)	0.966
Lymph metastasis	158/478 (33.1)	175/464 (37.7)	0.135	756/2092 (36.1)	923/2370 (38.9)	0.053
Distant metastasis	90/478 (18.8)	68/464 (14.7)	0.087	332/2092 (15.9)	380/2370 (16.0)	0.881
Liver metastasis	16/478 (3.3)	7/464 (1.5)	0.068	69/2092 (3.3)	82/2370 (3.5)	0.766
Bone metastasis	48/478 (10.0)	36/464 (7.8)	0.219	187/2092 (8.9)	192/2370 (8.1)	0.317
Brain metastasis	11/478 (2.3)	5/464 (1.1)	0.146	59/2092 (2.8)	72/2370 (3.0)	0.667
Lung metastasis	14/478 (2.9)	11/464 (2.4)	0.594	49/2092 (2.3)	76/2370 (3.2)	0.081
Bilateral	10/478 (2.1)	6/464 (1.3)	0.343	40/2091 (1.9)	45/2370 (1.9)	0.972
Chemotherapy	153/478 (32.0)	164/464 (35.3)	0.279	1403/2092 (67.1)	1627/2370 (68.6)	0.258
Hormone Therapy	410/478 (85.8)	390/464 (84.1)	0.460	1733/2092 (82.8)	1975/2370 (83.3)	0.660
Radiation therapy	75/478 (15.7)	67/464 (14.4)	0.592	708/2092 (33.8)	822/2370 (34.7)	0.555
Follow‐up time	478/478 (100.0)	464/464 (100.0)		2092/2092 (100.0)	2370/2370 (100.0)	
Median (IQR), months	40 (18, 74)	31 (16, 56)	< 0.001	45 (19, 83)	43 (18, 71)	< 0.001
Five‐year OS	85.0 (81.0–89.2)	79.3 (74.1–84.9)	0.405	94.9 (93.7–96.2)	94.6 (93.3–95.9)	0.576
HR negative						
*n* (%)						
Sex (male)	0/132 (0.0)	0/97 (0.0)	> 0.999	0/886 (0.0)	0/385 (0.0)	> 0.999
Age at diagnosis, *n*	132/132 (100.0)	97/97 (100.0)		886/886 (100.0)	385/385 (100.0)	
Median (IQR), years	70.0 (67.0, 74.5)	70.0 (67.0, 74.0)	0.992	49 (43, 55)	51 (46, 57)	< 0.001
Age ≥ 65 years	132/132 (100.0)	97/97 (100.0)	> 0.999	0/886 (0.0)	0/385 (0.0)	> 0.999
Post‐menopausal	132/132 (100.0)	97/97 (100.0)	> 0.999	273/497 (54.9)	133/201 (66.2)	0.006
Tumor size, *n*	118/132 (89.4)	88/97 (90.7)		820/886 (92.6)	347/385 (90.1)	
Median (IQR), cm	2.50 (2.00, 3.00)	2.00 (1.50, 3.50)	0.410	2.00 (1.50, 3.00)	2.00 (1.50, 3.00)	0.271
Tumor size ≥ 2.0 cm	92/118 (78.0)	59/88 (67.0)	0.080	542/820 (66.1)	232/347 (66.9)	0.801
Tumor stage III	24/120 (20.0)	18/87 (20.7)	0.903	138/832 (16.6)	69/352 (19.6)	0.212
KI67 ≥ 20	115/129 (89.1)	74/96 (77.1)	0.015	824/875 (94.2)	327/377 (86.7)	< 0.001
Grade 3	74/111 (66.7)	41/82 (50.0)	0.020	484/701 (69.0)	201/325 (61.8)	0.023
Lymph metastasis	55/132 (41.7)	29/97 (29.9)	0.068	292/886 (33.0)	145/385 (37.7)	0.105
Distant metastasis	33/132 (25.0)	17/97 (17.5)	0.176	189/886 (21.3)	85/385 (22.1)	0.766
Liver metastasis	6/132 (4.5)	4/97 (4.1)	> 0.999	26/886 (2.9)	18/385 (4.7)	0.119
Bone metastasis	18/132 (13.6)	3/97 (3.1)	0.006	98/886 (11.1)	37/385 (9.6)	0.441
Brain metastasis	7/132 (5.3)	5/97 (5.2)	0.960	50/886 (5.6)	24/385 (6.2)	0.680
Lung metastasis	14/132 (10.6)	6/97 (6.2)	0.242	56/886 (6.3)	25/385 (6.5)	0.908
Bilateral	8/132 (6.1)	2/97 (2.1)	0.197	25/886 (2.8)	10/385 (2.6)	0.822
Chemotherapy	87/132 (65.9)	57/97 (58.8)	0.269	798/886 (90.1)	343/385 (89.1)	0.597
Hormone Therapy	3/132 (2.3)	3/97 (3.1)	0.700	50/886 (5.6)	27/385 (7.0)	0.347
Radiation therapy	30/132 (22.7)	19/97 (19.6)	0.567	318/886 (35.9)	140/385 (36.4)	0.872
Follow‐up time	132/132 (100.0)	97/97 (100.0)		886/886 (100.0)	385/385 (100.0)	
Median (IQR), months	28 (12, 57)	35 (13, 73)	0.403	39 (16, 78)	39 (17, 71)	0.871
Five‐year OS	58.9 (48.1–72.2)	81.7 (72.8–91.6)	0.009	89.1 (86.4–91.8)	90.6 (86.9–94.5)	0.510

Abbreviations: ER, estrogen receptor; HER2, human epidermal growth factor receptor 2; HR, hormone receptor; IQR, interquartile range; *n*, total number; OS, overall survival; PR, progesterone receptor.

In the 5‐year overall survival rates were 94.1% for HER2‐low versus 93.3% for HER‐negative (*p* = 0.241) in the < 65 age group, and 80.1% versus 79.8% (*p* = 0.411) in the ≥ 65 age group for HER2‐low versus HER‐negative, respectively. In the HR‐positive group analysis, the 5‐year overall survival rates were 94.6% for HER2‐low versus 94.9% for HER‐negative (*p* = 0.576) in the < 65 age group, and 79.3% versus 85.0% (*p* = 0.405) in the ≥ 65 age group for HER2‐low versus HER‐negative, respectively. In the HR‐negative group analysis, the 5‐year overall survival rates were 90.6% for HER2‐low versus 89.1% for HER‐negative (*p* = 0.510) in the < 65 age group, and 81.7% vs. 58.9% (*p* = 0.009) in the ≥ 65 age group for HER2‐low versus HER‐negative, respectively (Table 2).

After comparing clinicopathologic features, we conducted Cox regression analyses to evaluate the prognostic differences associated with HER2‐low (Table 3). Due to the intensive co‐linearity among tumor size, lymphatic metastasis, and AJCC stage, only AJCC stage was included as the adjustment variable in our multivariate analysis. Similarly, between age ≥ 65 years and post‐menopausal status, we chose only age as the adjusting variable. Additionally, KI67 status, grade, hormone therapy, and chemotherapy were also included as adjusting variables; HR status was adjusted for the entire cohort, with details specified in the Table 3 notes. Hazard ratios (HRs) of HER2‐related overall survival were also analyzed (Table 3). In the < 65 age group, these HRs were not significant in the entire cohort (*p* = 0.241) or HR‐positive group (*p* = 0.577), remaining insignificant (*p* = 0.722 and 0.709, respectively) after multivariate adjustment. In the ≥ 65 age group, HER2‐related overall survival showed no significant differences in both the entire cohort (*p* = 0.414) and HR‐positive group (*p* = 0.403), remaining insignificant (*p* = 0.407 and 0.529, respectively) after multivariate adjustment. For the HR‐negative group age under 65, both univariate and multivariate analyses showed no significant differences. However, of particular clinical relevance, in the HR‐negative group aged ≥ 65, HER2‐low HRs for overall survival were significant (0.45, 95% CI: 0.24–0.83, *p* = 0.011) in univariate analysis and remained significant (0.34, 95% CI: 0.14–0.80, *p* = 0.014) following multivariate adjustment (Table 3).

**TABLE 3:** Hazard ratios of HER2‐low versus HER2‐negative in mortality of Breast cancer with respect to the Age at diagnosis.

Event	Entire cohort				Age ≥ 65 years				Age < 65 years			
	HR_1_ (95% CI)	*p*	Adjusted HR_1_	*p*	HR_1_ (95% CI)	*p*	Adjusted HR_1_	*p*	HR_1_ (95% CI)	*p*	Adjusted HR_1_	*p*
			(95% CI)^a^				(95% CI)^b^				(95% CI)^b^	
Entire cohort												
Overall survival	0.86 (0.71–1.03)	0.091	1.08 (0.74–1.57)	0.703	0.89 (0.66–1.19)	0.414	1.31 (0.69–2.50)	0.407	0.87 (0.69–1.10)	0.241	1.09 (0.68–1.76)	0.722
HR_2_ positive												
Overall survival	1.02 (0.82–1.27)	0.843	1.10 (0.83–1.46)	0.499	1.16 (0.82–1.62)	0.403	1.16 (0.74–1.82)	0.529	1.09 (0.81–1.45)	0.577	1.07 (0.75–1.53)	0.709
HR_2_ negative												
Overall survival	0.78 (0.55–1.11)	0.167	0.62 (0.39–0.98)	0.041	0.45 (0.24–0.83)	0.011	0.34 (0.14–0.80)	0.014	0.87 (0.57–1.32)	0.511	0.89 (0.52–1.51)	0.658

Abbreviations: HR_1_, hazard ratio; HR_2_, hormone receptor.

^a^ Adjusted for HER2 status, patient age at diagnosis, AJCC stage, KI67 status, grade, hormone therapy, and chemotherapy, HR status was also adjusted for the entire cohort.

^b^ Adjusted for HER2 status, AJCC stage, KI67 status, grade, hormone therapy, and chemotherapy, HR status was also adjusted for the entire cohort.

### 3.4 HER2‐Low and Age Play a Significant Prognostic Stratification Effect in TNBC

Patients were categorized into four groups based on HER2 status and age, as detailed in Table 4. In the analysis of the entire cohort, compared with the 5‐year overall survival 79.8 (75.7–84.1) of the HER2‐0 age ≥ 65 group, the overall survival was not significantly different in HER2‐low, significantly higher in patients only with age < 65 and those with both HER2‐low and age < 65, being 80.1 (75.5–84.9) (*p* = 0.410), 93.3 (92.1–94.5) (*p* < 0.001), and 94.1 (92.8–95.3) (*p* < 0.001), respectively. The corresponding insignificant HRs post‐multivariate adjustment were 0.79 (0.54–1.15) (*p* = 0.226), 0.29 (0.21–0.40) (*p* < 0.001), and 0.29 (0.21–0.41) (*p* < 0.001), respectively. For the entire cohort, HER2‐low did not show a difference in prognosis.

**TABLE 4:** Overall survival in various settings of HER2‐low and Age in Breast cancer.

Tumor groups	5‐year OS	*p* ^a^	Unadjusted HR_1_ (95% CI)	*p*	Adjusted HR_1_ (95% CI)^b^	*p*
Entire cohort						
Negative age ≥ 65	79.8 (75.7–84.1)	Reference				
Negative age < 65	93.3 (92.1–94.5)	< 0.001	0.27 (0.21–0.34)	< 0.001	0.29 (0.21–0.40)	< 0.001
Low age ≥ 65	80.1 (75.5–84.9)	0.410	0.88 (0.66–1.18)	0.395	0.79 (0.54–1.15)	0.226
Low age < 65	94.1 (92.8–95.3)	< 0.001	0.24 (0.18–0.31)	< 0.001	0.29 (0.21–0.41)	< 0.001
HR_2_ positive						
Negative age ≥ 65	85.0 (81.0–89.2)	Reference				
Negative age < 65	94.9 (93.7–96.2)	< 0.001	0.24 (0.18–0.33)	< 0.001	0.28 (0.18–0.43)	< 0.001
Low age ≥ 65	79.3 (74.1–84.9)	0.410	1.16 (0.82–1.62)	0.399	1.15 (0.74–1.81)	0.531
Low age < 65	94.6 (93.3–95.9)	< 0.001	0.26 (0.19–0.36)	< 0.001	0.30 (0.20–0.46)	< 0.001
HR_2_ negative						
Negative age ≥ 65	58.9 (48.1–72.2)	Reference				
Negative age < 65	89.1 (86.4–91.8)	< 0.001	0.25 (0.17–0.37)	< 0.001	0.32 (0.19–0.54)	< 0.001
Low age ≥ 65	81.7 (72.8–91.6)	0.009	0.44 (0.24–0.81)	0.009	0.29 (0.13–0.67)	0.004
Low age < 65	90.6 (86.9–94.5)	< 0.001	0.22 (0.14–0.36)	< 0.001	0.29 (0.16–0.54)	< 0.001

Abbreviations: HR_1_, hazard ratio; HR_2_, hormone receptor; OS, overall survival.

^a^ Calculated by the log‐rank test between the two groups.

^b^ Adjusted for AJCC stage, KI67 status, grade, hormone therapy, and chemotherapy, HR status was also adjusted for the entire cohort.

In the analysis of the HR‐positive group, compared with the 5‐year overall survival of 85.0 (81.0–89.2) in the HER2‐0 age ≥ 65 group, the overall survival was not significantly different in HER2‐low, significantly higher in patients only with age < 65, and those with both HER2‐low and age < 65, being 79.3 (74.1–84.9) (*p* = 0.410), 94.9 (93.7–96.2) (*p* < 0.001), and 94.6 (93.3–95.9) (*p* < 0.001), respectively. The corresponding insignificant HRs post‐multivariate adjustment were 1.15 (0.74–1.81) (*p* = 0.531), 0.28 (0.18–0.43) (*p* < 0.001), and 0.30 (0.20–0.46) (*p* < 0.001), respectively. In the HR‐positive group, no significant prognostic difference was observed for HER2‐low status.

In the analysis of the HR‐negative group, compared with the 5‐year overall survival of 58.9 (48.1–72.2) in the HER2‐0 age ≥ 65 group, the overall survival was higher in patients only with HER2‐low, significantly higher in patients only with age < 65 and those with both HER2‐low and age < 65, at 81.7 (72.8–91.6) (*p* = 0.009), 89.1 (86.4–91.8) (*p* < 0.001), and 90.6 (86.9–94.5) (*p* < 0.001), respectively. The corresponding significant HRs post‐multivariate adjustment were 0.29 (0.13–0.67) (*p* = 0.004), 0.32 (0.19–0.54) (*p* < 0.001), and 0.29 (0.16–0.54) (*p* < 0.001), respectively. For the HR‐negative group, HER2‐low demonstrated a prognostic difference in the age ≥ 65 group. This indicates that HER2 status and age provide valuable prognostic stratification in TNBC subgroups after multivariate adjustment.

**FIGURE 2:**
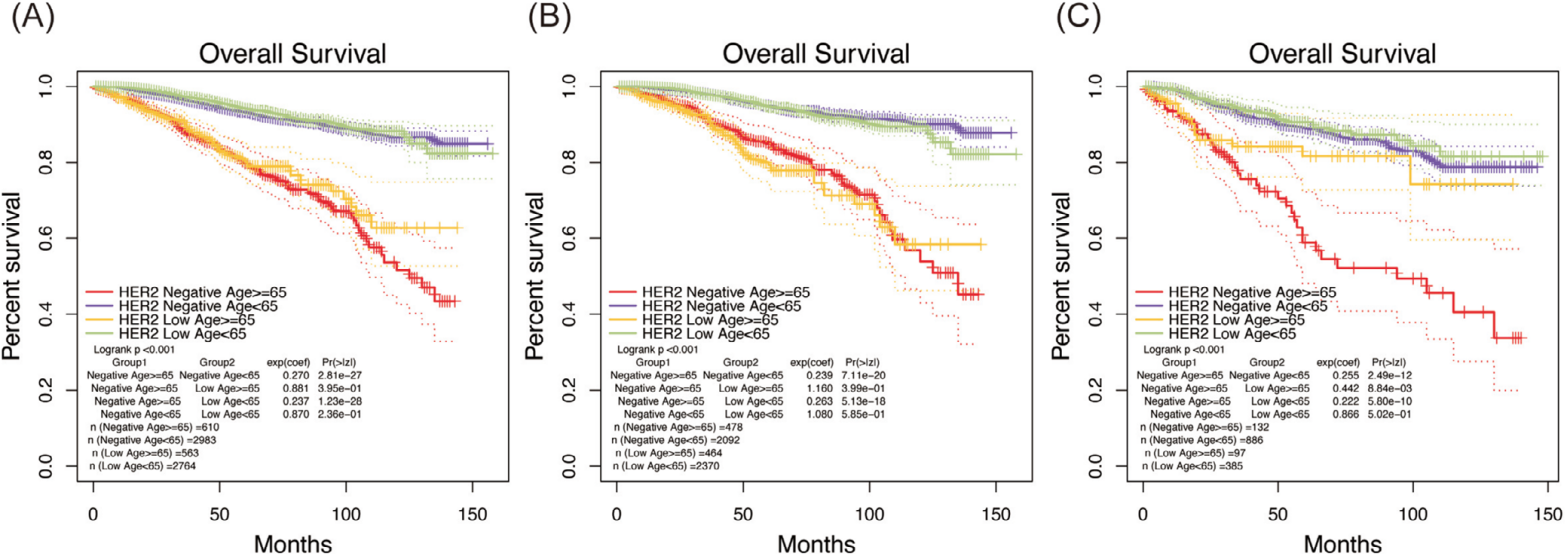
Kaplan–Meier analyses of patient survival probability associated with HER2 status and the impact of age on it in breast cancer. (A) Entire cohort. (B) HR‐positive. (C) HR‐negative.

Kaplan–Meier (K‐M) survival curve analysis was also conducted to further explore the impact of HER2‐low status and age on the overall survival of breast cancer. As depicted in Figure 2, the survival curves for both the entire cohort and the HR‐positive group showed no significant differences in HER2 status following age stratification. However, in the HR‐negative group aged ≥ 65, a significant prognostic disparity was observed between HER2‐low and HER2‐0 groups.

## 4 Discussion

Recent studies on antibody‐drug conjugate (ADC) agents have expanded treatment options for advanced metastatic HER2‐low breast cancer [10, 12]. The evolution from a dichotomous (HER2‐positive and HER2‐negative) to a trichotomous (HER2‐positive, HER2‐low, and HER2‐0) classification of HER2 status has undoubtedly facilitated personalized breast cancer treatment. Research on the HER2‐low phenotype has expanded beyond advanced breast cancer to encompass a broader patient cohort, including those with early‐stage breast cancer.

In this study, we first explored the clinicopathological differences between HER2‐low and HER2‐0 breast cancers. HER2‐low patients showed higher rates of ER, PR, and HR positivity, consistent with findings in almost all relevant studies [15, 18, 26, 27]. Generally, HER2‐low cancers demonstrated more favorable prognostic indicators. In the entire cohort, HER2‐low cancers showed lower rates of grade 3 (25.4% vs. 33.6%, *p* < 0.001) and bone metastasis (8.1% vs. 9.8%, *p* = 0.013, ‘metastasis’ here means those emerging during follow‐up). Within the TNBC subgroup, HER2‐low cancers demonstrated lower frequencies of Ki‐67 ≥ 20 (84.8% vs. 93.5%, *p* < 0.001) and grade 3 tumors (59.5% vs. 68.7%, *p* = 0.001). However, the opposite findings were presented in some subgroups, such as more lymph metastasis noted in HER2‐low in the entire cohort and Luminal type, and a greater percentage of KI67 ≥ 20 was found in Luminal.

We were particularly interested in the relationship between HER2‐low and age at diagnosis. A number of current studies of HER2‐low have shown the association between HER2‐low and the distribution of age. Horisawa et al. demonstrated among the 3541 HR‐positive patients, a lower proportion of patients age ≥ 65 was observed in HER2‐low patients (21.0% vs. 27.2%, *p* < 0.001), whereas among the 466 HR‐negative patients, a higher proportion of age ≥ 65 HER2‐low patients was observed (29.1% vs. 21.0%, *p* = 0.06) [24]. Tan et al. demonstrated among the 23,503 HR‐positive patients, a lower proportion of patients age ≥ 65 was observed in HER2‐low patients (12.3% vs. 13.6%), whereas among the 4634 HR‐negative patients, a higher proportion of age ≥ 65 HER2‐low patients was observed (17.1% vs. 12.5%) [19]. In a Chinese study, Han et al. demonstrated among the 2569 HR‐positive patients, a lower proportion of patients age ≥ 61 was observed in HER2‐low patients (30.2% vs. 23.9%, *p* < 0.001), whereas among the 1510 HR‐negative patients, a higher proportion of age ≥ 61 HER2‐low patients was observed (26.5% vs. 14.4%, *p* < 0.001) [19]. In another large Chinese study, Zheng et al. demonstrated among the 9902 HR‐positive patients, a lower proportion of patients age ≥ 60 was observed in HER2‐low patients (22.8% vs. 20.2%, *p* < 0.001), whereas among the 2009 HR‐negative patients, a higher proportion of age ≥ 60 HER2‐low patients was observed (21.6% vs. 15.9%, *p* < 0.001) [29]. Interestingly, our data also revealed that HER2‐low patients tended to be younger among HR‐positive patients while older in HR‐negative cases. Fewer HER2‐low patients had age ≥ 65 in HR‐positive (HER2 negative vs. HER2 low: 18.6% vs. 16.4%, *p* = 0.031), whereas in HR‐negative, HER2‐low patients had a significantly greater median age and a significantly greater proportion of patients with age ≥ 65 (13.0% vs. 20.1%, *p* < 0.001). Schettini et al. and Denkert et al. showed that in the entire cohort, HER2‐low patients had a greater proportion of elder patients [15, 18]. Besides, Jacot et al. showed that among the 296 TNBC patients, a higher proportion of patients aged ≥ 55 was observed in HER2‐low patients (68.7% vs. 53.2%, *p* = 0.047) [30]. Won et al. demonstrated among the 6930 HR‐negative patients, HER2‐low patients had a greater median age [50 (IQR 21–91) vs. 48 (20–91)] [26]. Numerous studies had documented the relationship between HER2‐low status and age distribution, particularly noting the older age of HER2‐low patients in TNBC. These findings align with our data, leading us to believe that the relationship between HER‐low and age distribution was not coincidental. Consequently, this had sparked our interest in thoroughly investigating the prognostic implications of age‐related HER2‐low status.

Although findings on age at diagnosis and prognosis of breast cancer may vary, many studies have shown that age is an important prognostic factor in breast cancer [31]. Several representative studies have found a “U” shaped relationship between age at diagnosis and breast cancer prognosis, indicating that both younger and older patients have poorer outcomes [32, 33, 34]. Several studies suggest that breast cancer patients diagnosed at a younger age (typically < 40 years) have a poorer prognosis [35, 36], and most research supports that older patients also face worse outcomes [37, 38, 39]. There is no universally accepted standard for defining “older” in the context of breast cancer, but 65 or 70 years is frequently used as the lower age limit [40]. The US National Comprehensive Cancer Network (NCCN) Breast Cancer Guidelines use 65 years as an age cutoff [41], and several studies also adopt 65 years as the threshold [42, 43, 44, 45]. Accordingly, we selected 65 years as the age cutoff in this study.

Utilizing the Kaplan–Meier (KM) method, univariate regression, and multifactorial correction, our study revealed no significant prognostic differences between HER2‐low and HER2‐0 across the entire cohort and in both HR‐positive and HR‐negative groups. Here, we do not discuss in detail the prognostic difference of HER2‐low at the overall level, as most current research, including ours, does not find significant differences in prognostic stratification for HER2‐low, as well as after correction for HR status [15, 22, 23, 24, 25, 26, 27]. This is consistent with the consensus at the ESMO meeting [16]. However, few studies have explored the prognostic differences of HER2‐low in more specific subgroups, as inspired by Mutai et al. who found that HER2‐low was significantly associated with better OS in a subgroup of patients with genomic risk (RS) over 25 in the Luminal type [28]. When categorizing by age at diagnosis with a 65‐year cut‐off, HER2‐low demonstrated a significant prognostic difference from HER2‐0 in TNBC aged ≥ 65 years. This suggests HER2‐low could be an independent prognostic factor in this specific subgroup. Following a four‐category grouping based on HER2 and age in TNBC, patients aged ≥ 65 with HER2‐0 showed significantly lower 5‐year survival compared to the other subgroups. This has crucial clinical implications, allowing for prognostic assessments based on HER2 status and age grouping in TNBC patients.

The notable prognostic difference between HER2‐low and HER2‐negative in TNBC patients aged ≥ 65 may be partly explained by clinicopathologic indicators. For instance, HER2‐low patients in this age group show lower rates of malignant markers like KI67 ≥ 20, Grade 3, and bone metastases. Ki‐67 positivity is associated with a higher risk of relapse and poorer survival outcomes in patients with early breast cancer [46]. Likewise, a higher histologic grade correlates with worse prognosis [47]. Metastasis is the underlying cause of death for the majority of breast cancer patients [48]. Although women with predominant or exclusive bone metastases generally have longer survival compared to those with metastases to other sites, the 5‐year overall survival rate is 22.8% [49]. Bone lesions often lead to severe and persistent complications, such as skeletal‐related events (SREs), which include pathological fractures, bone pain, hypercalcemia, and spinal cord compression. These complications significantly impact quality of life and can ultimately result in death [50, 51]. In a study by Sathiakumar et al. involving 7189 cases with bone metastases and 91,071 cases without bone metastases, the hazard ratios (HRs) for the risk of death were 4.9 (95% CI: 4.7–5.1) for women with bone metastases but without SREs and 6.2 (95% CI: 5.9–6.5) for women with both bone metastases and SREs, compared to women without bone metastases [52]. This may partly explain why there was a significant improvement in HER2‐low compared to HER2‐0 prognosis in the group with age ≥ 65.

This study had several strengths and weaknesses. Our dataset comprised 6920 HER2‐negative breast cancer patients, constituting a relatively large retrospective cohort for HER2‐low studies in the Chinese population. Our dataset includes an approximately 80% proportion of HR‐positive cases, similar to that observed in several large international HER2‐low studies. For instance, a study with NCDB data involving 1,136,016 participants, published in JAMA Oncology, reported an HR‐positive proportion of 86.4% [27]. Another study, involving 5235 participants and also published in this journal, reported an HR‐positive proportion of 86.7% [23]. This indicates that the HR‐positive rate in our cohort, while not excessively high, aligns with international findings. Additionally, our dataset includes 1500 TNBC cases, representing one of the larger cohorts in current Chinese HER2‐low studies. For the first time, we have combined insights from existing literature to explore the intriguing relationship between HER2‐low and age, and to delve deeply into the prognostic significance of HER2‐low. We examined the prognosis of HER2‐negative breast cancer not only at the overall level but also across various HR receptor statuses and age subgroups. This revealed the significant prognostic roles of age and HER2 status in TNBC subgroups, which are readily accessible markers, thus simplifying prognosis assessment in clinical practice. This study also has some limitations; as a single‐center study, there may be unknown bias. What's more, our dataset was exclusively composed of Chinese breast cancer patients, and due to racial and ethnic differences, our results might not apply to patients in other populations without further validation. So, our findings on HER2‐low and age require validation in broader and larger datasets. Furthermore, the lack of data on important gene mutation sites (like BRCA1/2, TP53) and gene expression profiles in our dataset limited our ability to thoroughly investigate the prognostic differences in HER2‐low within the age ≥ 65 subgroup in TNBC. Additionally, our dataset had a short follow‐up time of 41 months (IQR 18–73), potentially affecting prognosis assessments. We intend to extend the follow‐up period in future studies. Lastly, since HER2‐low primarily influences treatment decisions in advanced metastatic breast cancer, our results for early‐stage breast cancer may not align with findings in metastatic cases. Consequently, additional research is needed to explore the prognostic implications of HER2‐low expression and age in advanced metastatic breast cancer. Given these limitations, the results should be interpreted with caution.

## 5 Conclusions

In conclusion, our single‐center retrospective study of Chinese breast cancer patients revealed no significant prognostic differences between HER2‐low and HER2‐0 across the entire cohort, HR‐positive, and HR‐negative groups. For the first time, by integrating literature with our cohort data, we discovered and thoroughly analyzed the relationship between HER2‐low and age, revealing that HER2‐low patients tend to be older in HR‐negative cohort. HER2‐low status, in conjunction with age, may indicate a potential contribution to prognostic stratification in TNBC. Older (≥ 65) patients with HER2‐0 exhibited considerably poorer prognoses compared to other subgroups, underscoring a notable prognostic difference between HER2‐low and HER2‐0 in this older subset. Given the simplicity and accessibility of age and HER2 status as indicators, their stratification value in TNBC holds substantial clinical significance. We also expect this finding to be validated in larger and broader datasets, which could offer new insights into the prognostic assessment of breast cancer.

## Author Contributions

**Endong Chen:** funding acquisition (equal), investigation (equal), methodology (equal), writing – original draft (equal). **Chen Chen:** formal analysis (equal), investigation (equal), methodology (equal), software (equal), visualization (equal), writing – original draft (equal). **Yingying Chen:** supervision (equal). **Jie You:** data curation (equal), writing – review and editing (equal). **Nan Chen:** data curation (equal), writing – review and editing (equal). **Shenlin Xu:** formal analysis (equal). **Qingxuan Wang:** methodology (equal). **Yefeng Cai:** methodology (equal). **Xiaoqu Hu:** writing – review and editing (equal). **Quan Li:** conceptualization (equal), project administration (equal), writing – review and editing (equal).

## Ethics Statement

This retrospective population‐based cohort study, approved by the Institutional Ethics Committee of the First Affiliated Hospital of Wenzhou Medical University (Approval Number: KY2024‐288), utilized de‐identified data and adhered to the principles of the Declaration of Helsinki.

## Consent

The authors have nothing to report.

## Conflicts of Interest

The authors declare no conflicts of interest.

## Data Availability

The data used for these analyses cannot be shared by the authors for reasons of confidentiality.
